# Family satisfaction in the ICU: a 6-month experience

**DOI:** 10.1186/cc13217

**Published:** 2014-03-17

**Authors:** M Zouka, A Myrou, I Soultati, T Aslanidis, A Euthymiou, E Geka, E Anastasiou, V Ourailoglou, M Giannakou

**Affiliations:** 1 Papageorgiou General Hospital, Thessaloniki, Greece; 2 AHEPA University Hospital, Thessaloniki, Greece

## Introduction

The aim of the study was to prospectively assess family satisfaction in the intensive care setting and identify fields of possible improvement.

## Methods

The study was performed over a 6-month period in a 10-bed ICU. A properly translated and validated Family Satisfaction in the ICU (FS-ICU 24) questionnaire was used. A total of 126 questionnaires were handed out to family members upon patient discharge. The number of questionnaires returned for evaluation was 120 (91.5% participation). Five-point Likert scale responses were transformed to percentage scores, higher values representing better satisfaction.

## Results

Overall satisfaction with care was reported as very good or excellent by 81% of family members (32% and 49% respectively) both for ICU staff concern and caring as well as symptom management. Favorable scores were also noted in terms of decision-making, 89% of relatives reporting very good and excellent rates as regards information needs. On the other hand, most of the family members (73%) felt neither included nor excluded from the decision-making process. Moreover, a small number of them (6%) answered that they received poor emotional support. Finally, the frequency of communication with ICU nurses and the atmosphere in the waiting room were noted as fair by 28% and 47% of subjects respectively.

## Conclusion

Most family members were highly satisfied with ICU care their patients received. Nevertheless, our study showed that the fields of communication with the nurses as well as the waiting room atmosphere definitely need further improvement. Concerning emotional support and decision-making, the study revealed that currently used measures in our ICU still do not apply to all families and probably require a few changes.
